# Mastering Your Fellowship: Part 4, 2022

**DOI:** 10.4102/safp.v64i1.5593

**Published:** 2022-09-05

**Authors:** Mergan Naidoo, Andrew Ross, Tasleem Ras, Ts’epo Motsohi

**Affiliations:** 1 Department of Family Medicine, College of Health Sciences, University of KwaZulu-Natal, Durban, South Africa; 2 Department of Family Medicine, Faculty of Medicine, University of KwaZulu-Natal, Durban, South Africa; 3 Division of Family Medicine, Faculty of Medicine, University of Cape Town, Cape Town, South Africa; 4 Department of Family and Emergency Medicine, Faculty of Health Sciences, Stellenbosch University, Cape Town, South Africa

**Keywords:** family physicians, FCFP (SA) examination, family medicine registrars, postgraduate training, national exit examination

## Abstract

The ‘Mastering Your Fellowship’ series provides examples of the question format encountered in the written and clinical examinations, Part A of the Fellowship of the College of Family Physicians of South Africa examination. The series is aimed at helping family medicine registrars prepare for this examination.

## Introduction

This section in the *South African Family Practice* journal is aimed at helping registrars prepare for the Fellowship of the College of Family Physicians of South Africa (FCFP [SA]) Final Part A examination and will provide examples of the question formats encountered in the written examination: multiple choice question (MCQ) in the form of single best answer (SBA – type A) and/or extended matching question (EMQ – type R); short answer question (SAQ), questions based on the critical reading of a journal (evidence-based medicine); and an example of an objectively structured clinical examination (OSCE) question. Each of these question types is presented based on the College of Family Physicians’ blueprint and the key learning outcomes of the FCFP programme. The MCQs are based on the 10 clinical domains of family medicine, and the SAQs will be aligned with the five national unit standards. The critical reading section will include evidence-based medicine and primary care research methods.

This edition is based on unit standard 1 (critically reviewing new evidence and applying the evidence in practice, principles of self-care and leading a clinical governance team), unit standard 2 (evaluate and manage a patient according to the biopsychosocial approach) and unit standard 4 (facilitate the learning of others). The domain covered in this edition is anaesthetics. We suggest that you attempt to answer the questions (by yourself or with peers/supervisors) before finding the model answers online at http://www.safpj.co.za/.

Please visit the Colleges of Medicine website for guidelines on the fellowship examination: https://www.cmsa.co.za/view_exam.aspx?QualificationID=9.

We are keen to hear about how this series assists registrars and their supervisors in preparing for the FCFP (SA) examination. Please email us your feedback and suggestions (naidoom@ukzn.ac.za).

## Multiple choice question: Single best answer

A 32-year-old woman was rushed to the operating theatre after she presented with a ruptured ectopic pregnancy. Rocuronium was used for intubation and muscle relaxation. Post procedure, the muscle relaxant was reversed and the patient was extubated. The patient displayed jerky muscle movements and appeared anxious and distressed. Her oxygen saturation was normal, and the nerve stimulator exhibited two strong muscle twitches. What is the next most appropriate step?

- Administer face mask oxygen
- Administer midazolam
- Administer reversal agents
- Intubate the patient
- Reassure the patient

*Answer: c*

### Discussion

The patient displays symptoms and signs of residual neuromuscular blockade because of rocuronium. The non-depolarising muscle relaxants (NDMR), such as rocuronium, have a more prolonged onset of action and duration than depolarising muscle relaxants such as succinylcholine. Non-depolarisers compete with acetylcholine (ACh) by binding to and inactivating nicotinic receptors, preventing an action potential from being generated and causing muscle paralysis. Rocuronium, used in high doses, has a rapid onset of action and can be used as an alternative to succinylcholine. The higher doses lead to a longer duration of action and may result in prolonged muscle paralysis. Reversal agents consist of a combination of two drugs. The acetylcholine esterase (AChE) inhibitors (e.g. neostigmine) inhibit the enzyme that breaks down ACh, thus increasing the concentration of ACh. At the neuromuscular junction, competitive antagonism results in the non-depolarisers being replaced by the higher concentration of ACh at the nicotinic receptors and hence a return of muscle function. The AChE inhibitor causes an overall increase in ACh, affecting both nicotinic and muscarinic ACh receptors throughout the body. Activation of muscarinic receptors can result in bradycardia, bronchospasm, increased bronchial secretions, increased gut motility and increased gut secretions. An antimuscarinic agent (e.g. atropine or glycopyrrolate) is given together with the AChE inhibitor to counteract these effects.

The monitoring of the neuromuscular function is done clinically and with a nerve stimulator. Clinically evaluate if the patient can do the following:

- swallow and maintain a patent airway
- sustain a head lift for 5 s
- generate a negative inspiratory pressure of at least 25 cm H_2_O
- protrude their tongue, lift the arm and demonstrate handgrip strength

However, these measures are considered crude, and the recommendation is that a peripheral nerve stimulator is always available. The ulnar nerve is used most often for stimulation. The negative (black) electrode is placed over the ulnar nerve on the medial aspect of the wrist; the other positive (red) electrode is placed 2 cm proximal along the course of the ulnar nerve. The same electrodes used for the electrocardiogram (ECG) monitors are used and connected to the nerve stimulator leads. The nerve stimulator is switched on, and the train-of-four (TOF) button is pressed. This will deliver a current of 60 mA, causing the thumb to adduct and the little finger to flex.

Suppose the stimulus causes the little finger and thumb to twitch more than twice. In that case, it is evidence of the return of neuromuscular transmission, and the reversal drug (neostigmine and atropine or glycopyrrolate combination) can be given safely to reverse the effect of the relaxant. If the finger twitches less than twice, the neuromuscular block is still too intense, and the administration of a reversal drug will not have the desired effect. Take note that you should not use the same ulnar nerve more than twice for stimulation because the fatigue of the muscle can occur and cause less twitching. See Table 1 for the interpretation of TOF twitches.

**TABLE 1:** Twitch response and percentage of the blockade.

TOF response	Estimated percentage of receptors blocked by the NDMR
Four twitches	0–75
Three twitches	75
Two twitches	80
One twitch	90
No twitches	100

*Source*: Adapted from McGrath CD, Hunter JM. Monitoring of neuromuscular block. Cont Educ Anaesth Crit Care Pain. 2006;6(1):7–12. https://doi.org/10.1093/bjaceaccp/mki067 TOF, train-of-four; NDMR, non-depolarising muscle relaxants.

**FIGURE 1:**
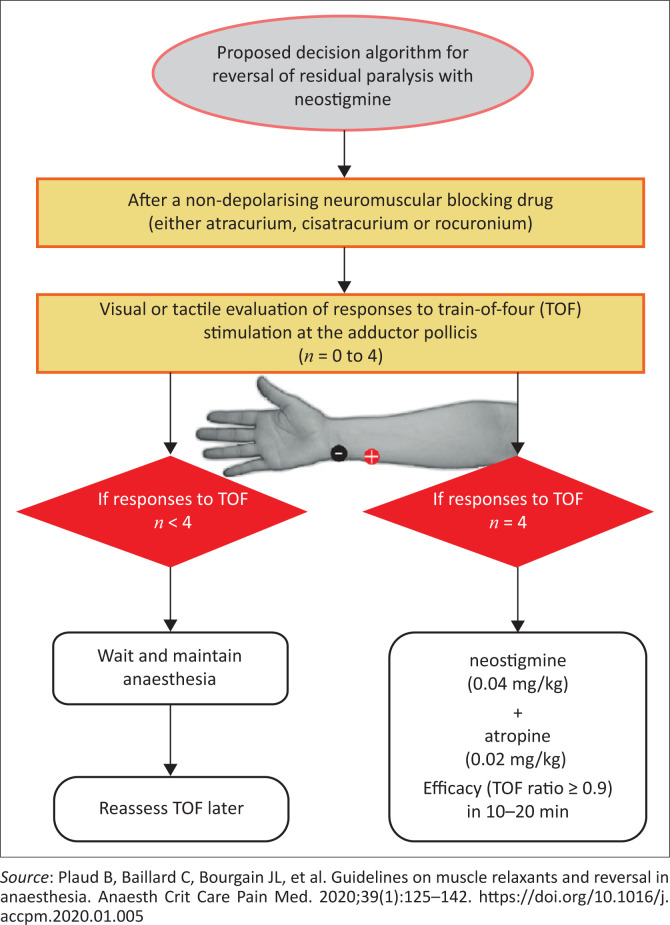
Decision algorithm for reversal using neostigmine.

Figure 1 provides a helpful decision tree to aid clinical judgment.

Features of inadequate reversal include jerky respiration, tracheal tug, poor chest expansion, poor cough, anxiety, weak handgrip and inability to raise the head from the pillow. The patient mentioned above displays residual neuromuscular blockade. Management involves administering one further dose of neostigmine with glycopyrrolate. If this is still insufficient, do not repeat but provide some sedation for an anxious patient. If ventilation is inadequate, this will need to be supported by manual or mechanical ventilation until a complete reversal occurs.

### Further reading

- Lombard A. Chapter 136: How to administer and reverse muscle relaxants. In: Mash B, Blitz J, editors. South African family practice manual. Braamfontein: Van Schaik; 2015, 447–449.
- Butterworth JF, Mackey DC, Wasnick JD. Neuromuscular blocking agents. Morgan and Mikhail’s clinical anesthesiology. 6th ed. New York, NY: McGraw-Hill Education; 2018.
- Plaud B, Baillard C, Bourgain JL, et al. Guidelines on muscle relaxants and reversal in anaesthesia. Anaesth Crit Care Pain Med. 2020;39(1):125–142. https://doi.org/10.1016/j.accpm.2020.01.005
- McGrath CD, Hunter JM. Monitoring of neuromuscular block. Cont Educ Anaesth Crit Care Pain. 2006;6(1):7–12. https://doi.org/10.1093/bjaceaccp/mki067

## Short answer question (SAQ): The family physician’s role as a leader and manager

You are the family physician working in a rural district hospital, which has 250 beds and is staffed with 12 medical officers (MOs; eight posts are vacant because of lack of funding). Approximately 300 deliveries are carried out a month in the hospital. Last month there was a maternal death because of a high spinal and failed intubation. The district and provincial assessors declared the death avoidable. Details of the case were caesarean delivery (CD) for foetal distress at 02:00. The patient was gravida 3 parity 2, with six antenatal visits. Clinically she appeared obese with a short neck.

Following the delivery of the spinal anaesthetic, the patient developed respiratory arrest. Neither the community service medical officer (CSMO) responsible for the anaesthetic nor the MO who was doing the CD could intubate or ventilate the patient, and the baby died because of hypoxic brain injury.

1. List and justify four issues that could have contributed to the poor outcome in this scenario. (4 marks)
2. What can you do to improve the individual capability of the doctors involved? (4 marks)
3. What can you do to improve the way things are organised in the facility to prevent such an event from recurring? (4 marks)
4. The CSMO is distraught at the outcome and is considering resigning and leaving medicine. As a family physician, how would you help the CSMO deal with this medical mistake? (8 marks)

Model answers

**1. List and justify four issues that could have contributed to the poor outcome in this scenario. (4 marks)**

- Inadequate skills mix in theatre with lack of training or competency of staff (one of the doctors on call should be able to give general anaesthesia for a high spinal).
- Inadequate pre-anaesthetic risk assessment of the patient (anticipation and preparation for difficult intubation – risk factors, such as obesity and short neck, need to be noted, in addition to the usual pregnancy risks for difficult intubation).
- Inadequate preparation of theatre – drugs and equipment in case of difficult intubation [difficult intubation trolley should be available with – different-sized endotracheal tubes ± introducer, gum elastic bougie (GEM) for difficult intubation, laryngeal mask anaesthesia (LMA), reversal drugs available following induction of anaesthesia (need to carefully consider the mode of anaesthesia before delivering the anaesthetic in high-risk cases, and not give long-acting muscle relaxants until the patient is safely intubated), bag and mask patient until help arrives].
- Inadequate process for calling for backup in an emergency (ideally should have backup for both CD and anaesthetic 24/7 with someone who can be in theatre within 10 min if the need arises).
- Shortage of staff (eight posts vacant), which increases pressure on staff and burnout.

**2. What can you do to improve the individual capability of the doctor(s) involved? (4 marks – *clinical governance activities that address doctors’ individual capability should be applied in this question; may include examples of capacity building or training or mentoring and risk management; assessment of competence of new clinical staff; mortality and morbidity (M&M) meetings and application of clinical guidelines*)**

- All staff need to be assessed for competency before being left to work independently. This can be performed by working alongside a senior doctor in theatre, receiving ‘bedside’ training and assessment, and keeping a skills logbook.
- Small group teaching or a skills lab session could be used to ensure training on pre-anaesthetic (airway assessment and management), anaesthetic (mode of anaesthesia and anaesthetic plan, as well as how to convert to general anaesthetic (GA) from spinal anaesthesia) and post-anaesthetic management.
- Bedside or small group teaching or skills lab should be used to train all staff for emergency scenarios – for example, converting to GA for a failed spinal, approach to the difficult airway, management of haemodynamic changes, and the use of vasopressors during a spinal anaesthetic for caesarean delivery (CD).
- Rigorous training in theatre preparation – ‘always be prepared’ – for example, anaesthetic machine check, appropriate equipment readily available and functioning, emergency drugs available and drawn up and ready for use. This includes easy access to a difficult intubation trolley with key equipment (endotracheal [ET] tubes with an introducer, GEM, Macintosh laryngoscope, laryngeal mask airway [LMA], etc.).
- Provide an implementation of clinical guidelines.
- Carry out regular M&M meetings so that things can be prevented from happening again.
- Arrange for additional anaesthetic training in the regional hospital or arrange regular visits from the anaesthetist on the district clinical specialist team (DCST).
- Encourage staff to call for help (even in the middle of the night). Provide mentoring and support to ensure that staff know that they can call for help when they suspect that a patient might have a difficult airway.

**3. What can you do to improve the way things are organised in the facility to prevent such an event from recurring? (4 marks – *clinical governance activities which address organisation in the facility should be applied in this question; may include activities that help to solve problems in teams, including root-cause analysis, including the use of a fishbone diagram and asking the ‘5 why’s’, audits and quality improvement cycles, improving patient safety and M&M meetings, revision of guidelines, and critically reviewing new evidence*)**

- Skills audit of MOs and accredit MOs with anaesthetic skills to the roster to ensure correct skills mix on the on-call roster.
- Ensure that a senior with anaesthetic experience is available 24/7 to provide additional support to those in theatre.
- Regular audits of emergency equipment and the difficult airway trolley with key equipment being available – ET tube with an introducer, GEM, Macintosh laryngoscope in theatre.
- Run regular drills on the management of the difficult airway.
- Ensure regular continuing medical education (CME)/training on airway management.
- Facilitate regular review of difficult anaesthetics with key learning outcomes with all staff – M&M meetings.

**4. The CSMO is distraught at the outcome and is considering resigning and leaving medicine. As a family physician, how would you help the CSMO to deal with this medical mistake? (5 marks)**

- Arrange a debriefing with the CSMO to understand what happened and the reasons for the decisions that were made.
- Allow for the ventilation of thoughts, emotions, and experiences associated with the event.
- Encourage CSMO to admit or acknowledge mistakes that were made and face the reality of what happened.
- Help them identify where they went wrong so as to prevent future error (use of the Gibbs reflective cycle might be a useful tool to encourage reflection and learning from this experience).
- Explore motives – was the CSMO trying to do the right thing at the time?
- Arrange professional counselling if necessary.
- Encourage them to talk to trusted peers and non-medical friends.
- Explore why they did medicine and explore their personal values, vision, and purpose.
- As the incident is serious, encourage the CSMO to immediately draft a statement, which factually sets out what happened and their involvement.
- Discuss the need for disclosure to the family.
- Any other appropriate responses.

### Further reading

- Medical Protection Society. Dealing with adverse incidents [homepage on the Internet]. 2020 [2022 May 30]. Available from: https://www.medicalprotection.org/uk/articles/dealing-with-adverse-incidents
- Couper I. Chapter 167. How to deal with a medical mistake. In: Mash B, Blitz J, editors. South African family practice manual. Braamfontein: Van Schaik; 2015, p. 572–573.
- Lundgren AC. Trends in maternal deaths associated with anaesthesia in the triennium 2017–2019. Obstet Gynaecol Forum. 2020;30(4):48–49.
- Rout CC, Farina Z. Anaesthesia-related maternal deaths in South Africa. S Afr J Anaesth Analg. 18(6):279–280. https://doi.org/10.1080/22201173.2012.10872868

## Critical appraisal of research

Read the accompanying article carefully and answer the following questions (*total 40 marks*). As far as possible, use your own words. Do not copy out chunks from the article. Be guided by the allocation of marks with respect to the length of your responses.

- Makoko UM, Modiba LM, Nzaumvila DK. Satisfaction with spinal anaesthesia for caesarean section at Tembisa Hospital, South Africa: A cross-sectional study. S Afr Fam Pract. 2019;61(2):39–47. https://doi.org/10.1080/20786190.2018.1531585

1. What was the aim of the study? (1 mark)
2. Identify three distinct arguments made by the authors to justify and provide a rationale for the study. (3 marks)
3. Explain why a quantitative research methodology may be most appropriate for this question. Comment on where and how a qualitative data collection methodology might still be applicable. (6 marks)
4. Critically appraise the sample size calculation. (3 marks)
5. Critically appraise how well the authors describe the validity of the data collection instrument or questionnaire. (5 marks)
6. Explain what linear regression means between age and perioperative explanation satisfaction scores in the study. (2 marks)
7. Critically appraise the comprehensiveness and appropriateness of the data collection and analysis. (10 marks)
8. The relationship between employment status and shivering was described as odds ratio (OR) 5.3; 95% confidence interval (CI) 1.9–14.1; *p* = 0.0009. What does this mean? (5 marks)
9. Odds ratios are used in this study. What do you understand by OR versus relative risk (RR)? (5 marks)

Total 30 marks

Model answers

**1. What was the aim of the study? (1 mark)**

To measure the level of satisfaction of mothers with spinal anaesthesia for caesarean section at Tembisa Hospital.

**2. Identify three distinct arguments made by the authors to justify and provide a rationale for the study. (3 marks)**

- Spinal anaesthesia remains the recommended type for caesarean sections and is the most commonly used procedure in district hospitals. Therefore, it is important to understand its many dimensions, including the patient’s perspective.
- Patient satisfaction is an essential aspect of the quality of healthcare that has not been assessed previously at Tembisa Hospital or similar hospitals in South Africa.
- Studies of patient satisfaction with spinal anaesthesia in African studies seem to demonstrate lower patient satisfaction rates than studies from the rest of the globe. Tembisa Hospital’s levels warrant measuring and comparing with the rest of the world.

**3. Critically appraise the authors’ decision to use a quantitative approach to answer the research question and comment on whether or not a qualitative approach might have been more appropriate. (6 marks)**

- A quantitative methodology in the form of a cross-sectional study with a self-administered survey questionnaire allows more participants to be included in the study. Previous studies have already determined the categories of care that patients consider important to consider. This is an area about which a lot is already known globally and on the continent. Therefore, a qualitative study exploring the different aspects of patients’ experiences of caesarean sections is arguably unnecessary. The authors are quite clear in which areas they require information about the patients’ experiences. This information is best collected quantitatively with discrete questions. The quantitative proportions (percentages) of patients recording their satisfaction or dissatisfaction with specific aspects of their care will assist better with building an argument for any changes that may need to be made. A quantitative study also allows for the collection of a wider range of aspects of care for these patients. Quantitative studies are typically more generalisable because of the larger number of cases they can cover. The data are also easier to aggregate, store, access and use. (3 marks)
- A qualitative study may still have value should there be a specific area of care upon which the researchers would like to focus in more depth and detail. For example, should patient satisfaction be unusually different from other studies in some aspects of preoperative care? Furthermore, exploring why multigravid patients seemed to be more satisfied with preoperative explanations is only speculated but not confirmed. These areas may warrant further in-depth qualitative exploration or even explanation with a qualitative study. (2 marks)
- In summary, a mixed-methods sequential study might have provided a more complete exploratory and explanatory picture, where some of the findings from the quantitative phases are explored in more depth by the qualitative phase. (1 mark)

**4. Critically appraise the sample size calculation. (3 marks)**

- The sample size of 82 women was calculated with a CI of 95% and a margin of error of 0.05. However, the authors do not indicate whether the calculation was made with the specific descriptive analyses. (1 mark)
- For a descriptive cross-sectional study, some use of satisfaction prevalence from other studies should be used as a reference in the calculation of sample size. This is not indicated in the study. The difference in categorical variables suggests an analytical aspect to this cross-sectional study. (1 mark)
- In such a study, the sample size calculation should factor in the minimum sample size required to be able to detect a difference between the categorical variables that will be compared using the Fisher’s exact analysis to which the authors refer. The important principle here is to be clear whether a cross-sectional study will be descriptive or analytical. Sample size calculation is dependent on the statistical analysis that will be performed. (1 mark)

**5. Critically appraise how well the authors describe the validity of the data collection instrument or questionnaire. (5 marks)**

- The authors state that a questionnaire that was validated in a previous study was used for this study after being modified. Validity refers to the extent to which an instrument measures what it purports to measure. This typically involves piloting and arguably should have been conducted if the questionnaire was modified and depending on the extent of the modifications. (2 marks)
- Justification for using questionnaires from previous studies is often advisable when reporting research and justification for why piloting was not performed. (1 mark)

There are different types of validity:

- Content validity refers to the relevance of each of the questionnaire’s items to the topic and the research aim.
- Face validity refers to how well participants answering the questionnaire understand the questions. In other words, how clear and logical are the questions to the participants?
- Construct validity refers to how an instrument measures the concept or theory it purports to measure.
- Criterion validity refers to how well a questionnaire correlates with another gold-standard questionnaire/tool that measures the same subject. (2 marks)

**6. Explain what is meant by linear regression between age and perioperative explanation satisfaction scores in the study. (3 marks)**

- This means an attempt was made to establish whether there was a statistically significant relationship between age and perioperative explanation satisfaction. As this relationship may be influenced by multiple other factors, these confounding factors are fixed or accounted for during the calculation using a linear regression model. (1 mark)
- The correlation coefficient (*r*) refers to the measurement of the strength between two variables. A linear correlation coefficient is a number calculated from given data that measures the strength of the linear relationship between these variables. The stronger the correlation between these two data sets, the closer it will be to +1 or –1. In this question, the two variables were age and perioperative explanation satisfaction. The correlation coefficient was weakly positive at 0.2, as shown in Figure 3. (2 marks)

**7. Critically appraise the comprehensiveness and appropriateness of the data collection and analysis. (10 marks)**

- Data collection was conducted comprehensively and managed equal representation of interviews of women during all three days of the post-caesarean section care in the hospital. However, there is no indication of how many of the patients refused to participate during the systematic random sampling process. This is important for considering selection bias. (1 mark)
- Furthermore, the number of incomplete questionnaires is not mentioned or discussed. This is an important aspect of assessing the true representativity of the study sample. (1 mark)
- The descriptive statistics included the proportions of various characteristics of the participants. These included demographic, gravidity, parity and caesarean section type (emergency versus elective) characteristics. These were presented adequately and comprehensively. However, there is a general shift to move away from racial classifications in research because of their unreliability and limitation of utility. (1 mark)
- Often, it is used erroneously where a more representative use of actual socioeconomic and geographical residence measurements for each case is more helpful and accurate for analysis. The racial terms are also used loosely and interchangeably with assumptions made by the authors for how they will be interpreted by the reader. Here, it is also important to assume that the reader will not only be a South African reader. (3 marks)
- Meaningful measurements of preoperative, intraoperative and postoperative levels of satisfaction are measured and presented. These proportions can be compared meaningfully to those in the literature from other regions. (1 mark)
- The authors proceed to compare proportions with various baseline characteristics using linear regression. For example, a statistically significant association was found between satisfaction with preoperative explanations and gravidity and employment. Similarly, there was a statistically significant relationship between the satisfaction with perioperative shivering and employment status. These results are difficult to use for meaningful interpretations and conclusions (inferences) mostly because the sample size calculations were not made with these multivariate comparisons in mind. (2 marks)
- The authors have fallen into the pitfall of trying to direct their study to analyse and conclude more than it was methodologically intended to do. In general, so-called post-hoc analyses and calculations, such as these should be conducted with caution. (1 mark)

**8. The relationship between employment status and shivering was described as OR 5.3; confidence interval (CI) 1.9–14.1; *p* = 0.0009. What does this mean? (5 marks)**

- This means that there was a strong association between shivering and a patient’s employment status, where employed patients experienced shivering more than unemployed patients. (1 mark)
- The strength of the association between being employed and shivering was strong because employed women in the group were 5.3 times more likely to shiver than those who were unemployed. The probability of this being because of chance alone is very low at 0.0009 and is significant because this probability is < 0.05. Lastly, we can be 95% confident that if this comparison were to be made numerous times with the same study design, the OR would fall between 1.9 and 14.1. This means that the OR would never be 1 because the CI does not cross 1. An OR of 1 indicates that there is no association. (2 marks)
- Clinically this is difficult to interpret and is not very meaningful as a univariate analysis which does not account for confounding variables as a multivariate (e.g. logistic regression) would do. The authors do expand on this a bit in the discussion section, mainly on measures on how to manage this in the postoperative period. But a logical explanation of how the employment status could explain this link is not provided. (2 marks)

**9. Odds ratios are used in this study. What do you understand by OR versus RR? (5 marks)**

- Both the OR and the RR are measures of the strength of association between a risk factor/exposure and an outcome (often a disease). However, their calculation differs slightly. (1 mark)
- The RR is calculated by dividing the probability of an outcome if an exposure/risk factor is present divided by the probability of an outcome if the exposure/risk factor is not present. With risk, the numerator is the number of times the outcomes occurred and the denominator is the total number of times the outcome could have occurred. Alternately, with the OR, the numerator is still the number of times that the outcome/disease occurred, but the denominator is the number of times the disease or outcome did *not* occur. (2 marks)
- The OR is usually a good approximation of risk, especially with rare conditions. It is also the only way to calculate risk in case–control studies, where the selected number of exposed and non-exposed individuals does not always reflect the natural frequency of the disease but is determined by the researcher. (1 mark)
- Calculating the OR alongside the RR for randomised clinical trials and cohort studies allows one to be able to compare the ORs to those of case controls of the same topic. This is often useful in the meta-analyses of different study designs. (1 mark)

### Further reading

- Pather M. Chapter 11: Evidence-based family medicine. In: Mash B, editor. Handbook of family medicine (4th edn.). Cape Town: Oxford University Press; 2017:​430–453.
- Riegelman R. Studying a study and testing a test. 5th ed. Philadelphia, PA: Lippincott Wiliams & Wilkins; 2005.
- Goodyear-Smith F, Mash B, Editors. How to do primary care research. Boca Raton, FL: CRC Press, Taylor and Francis Group; 2019.

## Objectively structured clinical examination (OSCE) scenario

### OSCE station

Anaesthesia.

### Objective of the station

This station tests the candidate’s ability to teach an intern how to conduct a pre-anaesthetic risk assessment.

### Requirements

- Simulated patient: young man/woman – intern
- Torchlight to visualise oral cavity
- Stethoscope
- Chest X-ray (CXR) of a patient with mitral stenosis
- ECG of a patient with mitral stenosis

### Instructions for the candidate

You are the family physician working in a rural district hospital. Before your elective caesarean section list, you decide to examine the patient quickly. You ask the intern who had clerked the patient to present the next patient on the list.

### Your task

Engage with this intern about the patient you see together. You do not need to examine this patient. All examination findings will be provided on request.

### Instructions for the examiner

This is an integrated consultation station in which the candidate has 15 min. Familiarise yourself with the assessor guidelines, which detail the required responses expected from the candidate.

No marks are allocated. In the mark sheet, tick off one of the three responses for each of the competencies listed. Make sure you are clear on what the criteria are for judging a candidate’s competence in each area.

### Further reading

- Klocke M. How to do a pre-anaesthetic consultation. In: Mash B, Blitz J, editors. SA family prac manual. 3rd ed. Pretoria: Van Schaik, 2015; p. 422–425.

### Guidance for the examiners

**FIGURE 2:**
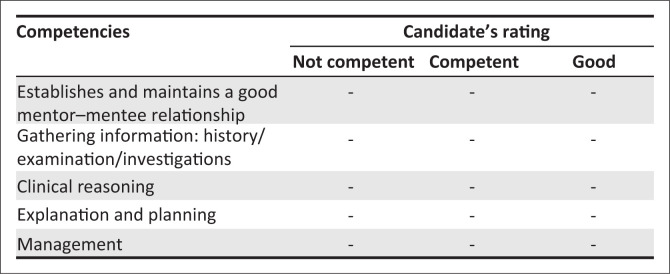
Marking template for consultation station.

The aim is to establish that the candidate can teach an intern how to conduct a pre-anaesthetic risk assessment (Figure 2).

*Competent: the task is completed safely and effectively*.

*Good: In addition to displaying competence, the task is completed efficiently and in an empathic, patient-centred manner (acknowledges patient’s ideas, beliefs, expectations, concerns/fears)*.

- Establishes and maintains a good mentor–mentee relationship
  - The competent candidate allows the intern to express themselves, showing respect at all times. The good candidate is engaging, patient, and kind.
- Gathering information
  - The competent candidate gathers sufficient information to establish a diagnosis of cardiac pathology with cardiac failure (*medical history, risk factors, current symptoms, soft diastolic murmur of mitral stenosis, with signs of cardiac failure P-wave abnormalities on ECG and pulmonary congestion on CXR*), though may not be able to make a specific diagnosis. This candidate also establishes the intern’s baseline knowledge and skill level.
    The good candidate identifies specific signs and findings to make a specific diagnosis of mitral stenosis with cardiac failure (*p-mitrale in affected leads and a detailed description of CXR abnormalities*) and gathers sufficient information to clearly delineate the knowledge/skills gap in the intern.
- Clinical reasoning
  - The competent candidate identifies the possible diagnosis (though may not be exact) of a cardiac lesion, with underlying cardiac decompensation, and consequent high risk, needing referral to a regional hospital for an elective caesarean section (CS). She or he also identifies that the intern is not yet safe in this domain.
  The good candidate makes a specific diagnosis of mitral stenosis with cardiac decompensation and recognises the need for referral. She or he additionally explores the intern’s reaction to this incorrect assessment.
- Explaining and planning
  - The competent candidate clearly explains the reasons for classifying this patient as high risk and referring her or him to the regional/tertiary hospital. She or he also checks that the intern understands this and elicits questions.
  The good candidate additionally uses a standardised framework (Pendelton or agenda led outcomes based analysis [ALOBA]) to provide feedback to the intern.
- Management
  - The competent candidate instructs that the referral to a higher level of care will take place and has a structured plan in place to ensure that this happens timeously. Resources are provided to the intern to learn about the pre-anaesthetic risk assessment.
  The good candidate additionally negotiates a semi-structured learning plan with the intern around the knowledge/skills gaps and ensures that a follow-up activity is in place to address these gaps.

## Role-play: Instructions for the actor

Young man/woman. Calm. You are an intern in a district hospital, joining your consultant on the elective caesarean section list. You have clerked the next patient and presented her to the consultant.

### Opening statement

‘The next patient for elective caesarean section was booked because she has a soft cardiac murmur. I’m not sure what the problem is, but she seems stable.’

### Open responses

Freely tell the doctor if asked …

This is her first pregnancy. She is 36 weeks by date. No antenatal problems thus far.

In the last three weeks, she reported swelling of her legs and progressive shortness of breath. You think this is because of normal late pregnancy.

You did your anaesthesia block at the tertiary hospital. You can manage airways and monitor patients in general anaesthesia. The anaesthetic registrar was supposed to teach you, but you did not learn much – it was the middle of the pandemic, and elective surgery was cancelled.

### Closed responses

Only tell the doctor if she or he brings this up.

Provide the examination findings.

Show the ECG and CXR: ‘It looks normal to me.’

You think this patient has a heart problem, but as she does not have heart failure, the risk is low. Also, a caesarean section is a quick operation, so this would also minimise risk.

You have never been shown how to do a preoperative risk assessment and had minimal obstetric anaesthesia exposure before.

### Ideas, concerns and expectations

You are shocked to hear that you had made such a significant mistake.

Will this mistake affect your signing off?

You are willing to learn to protect patients in the future.

#### Patient’s notes/examination findings

- Shallow tachypnoea with a respiratory rate of 23 breaths/min
- Bilateral pedal oedema (++)
- Chest: crackles at both lung bases
- Cardiovascular system (CVS): Heart rate of 96 beats/min
   Diastolic murmur of low pitch, rumbling in character at the apex
- CXR: Enlarged heart shadow
   Straightening of the left cardiac border
   Prominent pulmonary vessels
   Presence of Kerley B lines
- ECG: Bifid P-waves (wide > 0.11 s) in leads I, II and aVL
- Weight: 72 kg
- Height: 1.57m
- Blood pressure: 130/80 mmHg
- Haemoglobin: –9.4 g/dl
- Serum creatinine: 85 μmol/L (ref 49–90)
- Urea: 6.5 mmol/L (ref < 8.4)
- Random blood glucose: (HGT) 8 mmol/L
- Urinalysis: protein (trace)

**FIGURE 3:**
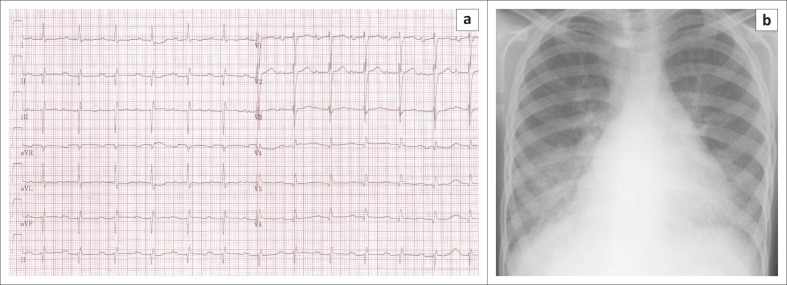
Electrocardiogram (3a) and chest X-ray (3b).

Refer to Figure 3a for patient electrocardiogram and X-Ray (Figure 3b).

